# The Role of Polyploidy in the Genetic Structure and Expansion of *Lepisorus clathratus* in the Qinghai–Tibetan Plateau and Hengduan Mountains

**DOI:** 10.3390/plants13223181

**Published:** 2024-11-13

**Authors:** Cunfeng Zhao, Xianchun Zhang

**Affiliations:** 1 Chongqing Institute of Green and Intelligent Technology, Chinese Academy of Sciences, Chongqing 400714, China; zhaocunfeng@cigit.ac.cn; 2 State Key Laboratory of Systematic and Evolutionary Botany, Institute of Botany, Chinese Academy of Sciences, Beijing 100093, China

**Keywords:** polyploidy, genetic structure, gene flow, post-glacial expansion, ferns, Qinghai–Tibetan Plateau, Hengduan Mountains

## Abstract

Polyploidy plays a crucial role in plant evolution, particularly in shaping genetic diversity and geographic distribution. This study investigates the genetic diversity and distribution of *Lepisorus clathratus* (C. B. Clarke) Ching, a polyploid fern species endemic to the Qinghai–Tibetan Plateau and Hengduan Mountains. We sampled 586 individuals from 66 populations and identified three ploidy levels: diploid, tetraploid, and hexaploid. Flow cytometry and chloroplast DNA sequencing were used to assess ploidy variation and genetic structure. Tetraploid populations dominated the Hengduan Mountains and exhibited wider geographic ranges, while diploids were largely confined to the Qinghai–Tibetan Plateau. Molecular variance analysis revealed significant genetic differentiation among regions, with polyploid populations demonstrating higher cross-region migration rates compared with diploids, as evidenced by the historical gene flow analysis. Ecological niche modeling suggested that polyploids expanded more successfully in post-glacial periods, likely due to their greater ecological flexibility and capacity for long-distance colonization. These findings highlight the critical role of polyploidy in shaping genetic structure and species expansion, contributing to the understanding of plant adaptation in response to historical climatic changes.

## 1. Introduction

Plant populations exhibit intricate genetic distribution patterns that are influenced by a variety of evolutionary processes. Among these, polyploidy—an increase in the number of chromosome sets—plays a crucial role in shaping the genetic and geographic structure of species [1]. Polyploidy can result in immediate reproductive isolation and can facilitate rapid adaptation to new environments, thus promoting diversification and speciation [2,3]. Polyploidy is particularly common in plants and has significant implications for their geographic distribution and evolutionary history. Polyploid individuals often exhibit greater ecological tolerance and can colonize a wider range of habitats compared with their diploid counterparts [4,5]. This ecological flexibility enables polyploids to establish and spread in new areas more effectively, contributing to the complex geographic patterns observed in many plant species [6,7]. For example, polyploids are often found in regions with extreme environmental conditions, where diploids are less competitive [8]. Understanding the genetic differentiation of plant populations requires examining the geographic distribution of chromosomal ploidy levels and how these patterns influence gene flow and population expansion.

The Quaternary period, marked by repeated glaciation events, profoundly affected the distribution and genetic composition of plant species. These glacial cycles caused range contractions and expansions, creating opportunities for genetic differentiation and polyploid establishment [9,10]. During glacial maxima, populations were often confined to refugia—small, isolated areas with suitable climatic conditions—leading to genetic bottlenecks and divergence. As the ice sheets receded during post-glacial periods, these plants expanded their ranges beyond refugial areas, encountering new environments and often hybridizing with other populations. This process sometimes led to polyploidization [11,12]. The ability of polyploids to colonize new niches and their subsequent reproductive isolation from diploid progenitors create a dynamic landscape of genetic diversity [13]. Polyploids can act as a bridge for gene flow between populations that would otherwise remain isolated, thus playing a critical role in the evolutionary history of plant species [14]. The resilience of polyploids to environmental changes suggests that they may play a crucial role in the response of plant species to ongoing climate change [5]. The advance and retreat of ice during the Quaternary glaciations provides a unique context for studying the interplay between polyploidy and plant population dynamics. This post-glacial expansion, documented in several plant groups, provides insights into how historical climate events have shaped current genetic diversity patterns [10,15].

The Qinghai–Tibetan Plateau and the Himalayan–Hengduan Mountains regions are important biodiversity hotspots covering the Qinghai–Tibetan Plateau sensu stricto (QTP*ss*), the Himalayas, and the Hengduan Mountains (HDM) and are characterized by unique geographic distributions and complex migration patterns [16]. These regions have undergone significant climatic and geological changes that influenced the dispersal and genetic differentiation of plant species [17]. The uplift of the Qinghai–Tibetan Plateau and the formation of the Hengduan Mountains created diverse microhabitats, facilitating speciation and migration [18]. During interglacial periods, plant populations migrated vertically along elevation gradients to suitable habitats, contributing to high endemism and genetic diversity [19]. Post-glacial recolonization further shaped the current distribution patterns, with many species exhibiting fragmented distributions across these regions [20]. These complex biogeographic processes highlight the importance of the Qinghai–Tibetan Plateau, the Himalayas, and the Hengduan Mountains in understanding plant evolution and diversity.

*Lepisorus clathratus* (C. B. Clarke) Ching (Polypodiaceae) is an endemic alpine fern species mainly found in the Himalayan–Hengduan Mountains regions. Its genetic diversity and distribution are significantly influenced by Quaternary climate changes [21]. Glacial cycles induced range contractions and expansions, shaping the genetic structure observed in contemporary populations. Despite the prevalence of polyploidy in ferns, studies on the relationship between polyploidy and population genetics and distribution patterns remain scarce, particularly in the alpine regions of southwestern China. In this study, we investigate the genetic distribution patterns of plant populations with varying ploidy levels across different geographic regions of *L. clathratus*. We focus on how polyploidy contributes to population expansion and the establishment of new populations, particularly in the context of the Quaternary glaciation. By examining these processes, we aim to elucidate the mechanisms underlying plant population differentiation and the role of polyploidy in shaping contemporary plant diversity.

## 2. Results

### 2.1. Geographical Distribution of Different Ploidy Levels

**Figure 1:**
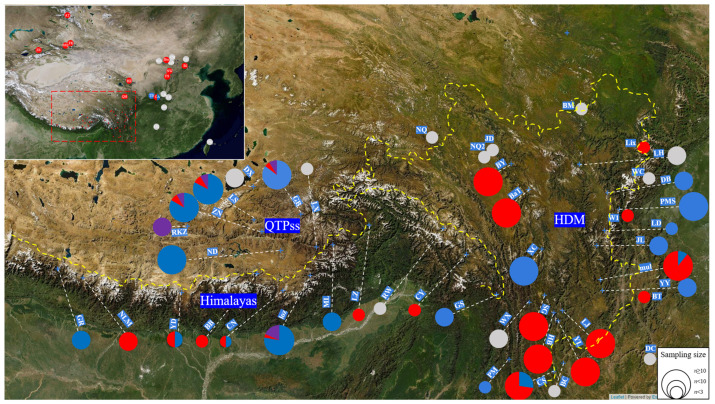
Sampling locations and the chromosome ploidy levels of different populations. The circles in this figure represent the populations, with their sizes indicating the number of individuals within each population. The pies within these circles depict the composition of chromosome ploidy levels, distinguished by color: blue for diploids, red for tetraploids, purple for hexaploids, and gray-white for those with unknown ploidy levels. The dashed red box in the top-left corner highlights the focal region. Meanwhile, the yellow dashed line in the bottom-right corner delineates the boundaries of the three focal regions: the Qinghai–Tibetan Plateau sensu stricto (QTP*ss*), the Hengduan Mountains (HDM), and the Himalayas.

We analyzed a comprehensive dataset of 586 *Lepisorus clathratus* individuals sourced from 66 populations across its distribution range in China. Of these, the ploidy level was determined for 448 individuals from 45 populations, revealing three distinct ploidy levels: diploid, tetraploid, and hexaploid (Figure 1, Table S1). The tetraploid and diploid specimens were the most prevalent, accounting for 52.46% and 43.75% of the total individuals, respectively, while hexaploids comprised only 3.79% of the samples. Among the populations with determined ploidy data, there were 23 tetraploid populations, 12 diploid populations, and only 1 hexaploid population. There was a total of nine mixed-ploidy populations, with five diploid–tetraploid mixed populations and four diploid–tetraploid–hexaploid mixed populations.

Significant regional variations were observed in the geographical distribution of populations with different ploidy levels. Tetraploids were primarily distributed in the Hengduan Mountains, northwestern China, northern and central regions, as well as Taiwan of China. Diploid populations predominantly occupied the QTP*ss*, while diploid and tetraploid populations were roughly equal in numbers in the Himalayan region. In the Hengduan Mountains, tetraploids were relatively more numerous and more spatially distributed compared with diploids, although there were differences between the eastern and western regions. Among the 18 populations with ploidy data, there were 9 tetraploid populations, 7 diploid populations, and 2 mixed-ploidy populations, which were dominated by tetraploids. The populations in the QTP*ss* were primarily diploid. Among the five populations with ploidy data, there was one diploid population, one smaller hexaploid population, and three mixed-ploidy populations dominated by diploids, which contained a very small proportion of tetraploid and hexaploid individuals. In the Himalayan region, diploid and tetraploid populations were roughly equivalent. Among the 10 populations with ploidy data, there were 3 diploid populations, 4 tetraploid populations, and 3 mixed-ploidy populations. The latter included two populations with equal proportions of diploids and tetraploids, and one larger population in which diploids dominated and which had a few hexaploids and even fewer tetraploids.

### 2.2. Chloroplast DNA Sequence Data and Phylogenetic Structure

**Figure 2:**
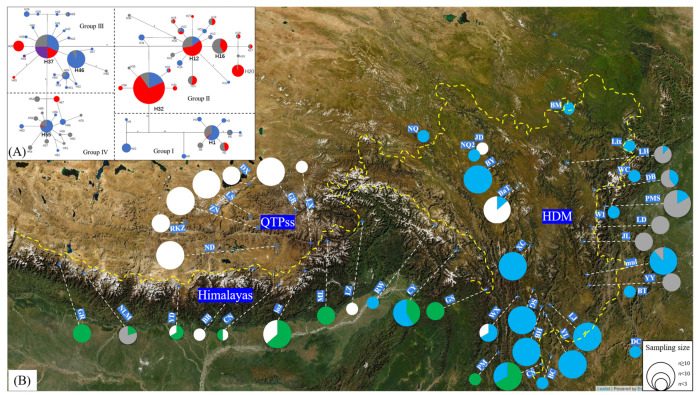
Haplotype network and composition of the haplotype groups in *Lepisorus clathratus* populations within the focal regions. (**A**) Haplotype network and group division: This panel displays the haplotype network of all sampled *L. clathratus* individuals, along with the division into haplotype groups (Group I to IV). The color of the pies within the network indicates the ploidy level of each haplotype: blue for diploids, red for tetraploids, purple for hexaploids, and gray-white for haplotypes with unknown ploidy levels, with their sizes indicating the number of individuals within each. (**B**) Population-specific haplotype group composition: The size of the circles represents the number of individuals in each population, and the color of the pies indicates the haplotype group: green for Group I, blue for Group II, white for Group III, and gray for Group IV.

We obtained *matK*, *psbZ*, *rpl32-trnP*, and *trnT* sequences from all 586 individuals and from the outgroup *Lepisorus scolopendrium* (Ching) Mehra & Bir. The length of the concatenated chloroplast DNA (cpDNA) was 2750 bp, which contained 133 polymorphic sites with 51 parsimony informative sites. In total, 70 haplotypes were identified (Figure 2): 15 haplotypes in the Himalayas, 19 in QTPss, 34 in HDM, and 13 haplotypes in the non-focal region (Table 1 and Table S1). There were 20 haplotypes shared by two or more populations, with the most common being H32, H37, H12, H46, H1, H55, and H16. Four haplotypes (H12, H16, H21, H22) were shared between the focal region and the non-focal region of this study. There were 21 populations containing at least one private haplotype. Among them, 10 populations were distributed in the HDM, four populations in the Himalayan region, three populations in the QTP*ss*, and four populations located in the non-focal region. In the focal region, HDM had 19 private haplotypes, with 8 in the western Hengduan Mountain region (WHDM) and 11 in the eastern Hengduan Mountain region (EHDM). QTP*ss* had 15 private haplotypes, and the Himalayas had 9 private haplotypes. The populations with a higher number of private haplotypes were GB, LS, and ND located in QTPss; LH and PMS in EHDM; SG in WHDM; loz in the Himalayas; and TB in the non-focal region.

The MJ network analysis and phylogenetic trees yielded similar topologies, with both dividing the samples into four groups (Figure 2A and Figure S1). This phylogenetic division did not fully align with the ploidy structure across the distribution range, as sympatric diploids and tetraploids often clustered within the same group. Group I, composed of haplotypes H1–H10, exhibited a distinct geographical distribution, primarily in the Himalayan region, with a few individuals in the populations (PM and CS) on the western edge of the HDM (Figure 2B). The distribution area of Group II (including haplotypes H11–H27 and H30–H36) was the most extensive, encompassing the entire Hengduan Mountains and the eastern edge of the Himalayas, and, more widely, areas of northern China, northwestern China and Taiwan (Figure S2). Group III (H28–H29, H37–H53, and H68–H69) mainly consisted of specimens from the QTP*ss*, with a few individuals found in the Himalayas and the western edge of the HDM. Group IV (H54–H67 and H70) was predominantly composed of specimens from the EHDM, with a few samples from the NLM population in the Himalayas. The populations from the QTP*ss* exhibited a relatively consistent genetic structure, with all specimens belonging to Group III. Populations of the non-focal region of this study also similarly exhibited this consistent genetic structure, with all individuals belonging exclusively to Group II. In the Himalayas, the genetic structure both within and between populations showed significant variability, exhibiting distinct characteristics of divergence: while more than half the samples were grouped in Group I, others belonged to Groups II, III, and IV. Notably, latter plants from the other groups were all polyploid. A similar pattern was observed in the HDM populations, where approximately half of the samples in the eastern HDM were assigned to Group IV, while the other half belonged to Group II, with most being polyploid. In the western HDM, approximately 80% of the plants belonged to Group II, while the remaining plants were roughly evenly divided between Group I and Group III, with most being polyploid.

### 2.3. Genetic Diversity and Population Structure

Among the three focal regions, the Himalayan region exhibited the lowest number of haplotypes but demonstrated the highest levels of haplotype diversity and nucleotide diversity (*h* = 15, *Hd* = 0.8418, π = 0.00524; Table 1). In contrast, the QTP*ss* region, despite having a higher number of haplotypes (*h* = 19), displayed the lowest nucleotide diversity (π = 0.00053). The HDM region had the greatest number of haplotypes (*h* = 34), although it exhibited moderate levels of haplotype and nucleotide diversity (*Hd* = 0.7489, π = 0.00265). Notably, the western and eastern parts of the HDM (WHDM and EHDM) had approximately equal numbers of haplotypes and exhibited comparable genetic diversity (WHDM: *h* = 19, π = 0.00224; EHDM: *h* = 20, π = 0.00249).

Significant genetic differentiation was revealed among the populations of *Lepisorus clathratus* based on four cpDNA fragments, as indicated by the AMOVA analysis (Table 2). Genetic differentiation was observed both among different regions and across ploidy levels, with their fixation indices showing statistical significance (*p* < 0.001). However, geographic regions contributed considerably more to the population structure, with nearly 30% of the genetic variation partitioned among regions, compared with only about 6% among ploidy levels. Genetic variation within populations across ploidy levels was low, with non-significant *p*-values (*p* > 0.05).

### 2.4. Demographic History and Gene Flow Between Populations

**Figure 3:**
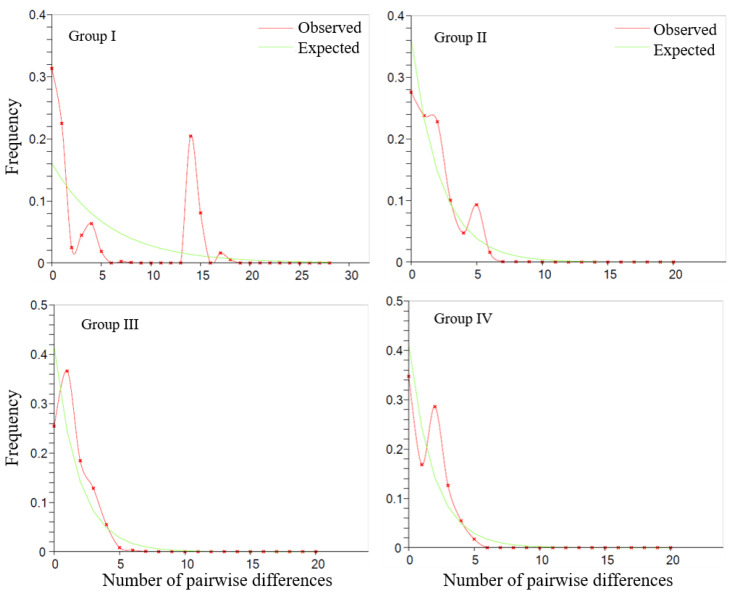
Mismatch distribution analysis of each haplotype group for *Lepisorus clathratus*. The division of haplotype groups follows the classification shown in Figure 2A.

The demographic histories of the different *Lepisorus clathratus* population groups varied. For Group I and Group II, no evidence of demographic expansion was detected, as indicated by the multimodal mismatch distribution patterns (Figure 3) and non-significant values for Fu’s *Fs* and Tajima’s *D* indices (*p* > 0.05) (Table 3). Consequently, the hypothesis of demographic expansion was rejected for these groups. However, there were certain differences between the two, as the latter had a less multimodal distribution than Group I. This difference was also reflected in the network results, where Group II exhibited a more star-like shape. In contrast, Group III and Group IV exhibited clear evidence of expansion, with unimodal mismatch distribution patterns and significant Fu’s *Fs* and Tajima’s *D* values in the neutrality tests (*p* < 0.05). Both groups showed the star-like shape observed in the network results (Figure 2A).

**Figure 4:**
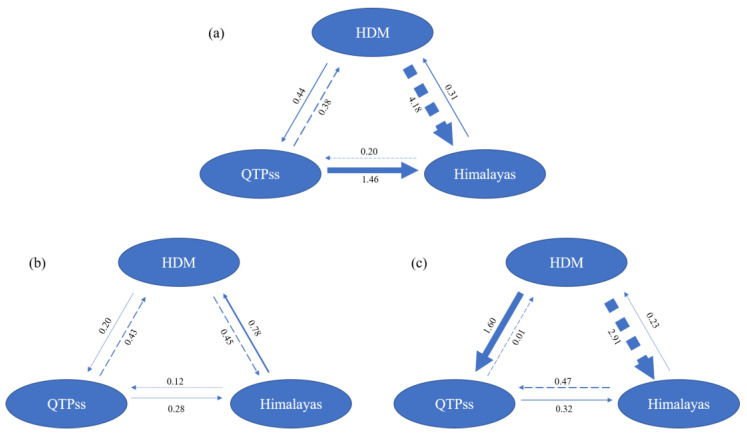
Estimates of historical gene flow using MIGRATE v.4.4.3 within (**a**) the historical gene flow based on all the samples, (**b**) the historical gene flow based on diploid samples, and (**c**) the historical gene flow based on polyploid samples. Dashed line arrows are utilized to clearly distinguish them from solid line arrows. These two types of arrows represent opposite directions of gene flow. The data units displayed on the arrows are individuals per generation, which can be interpreted as the number of individuals that migrate into a population in each generation.

We used MIGRATE to infer the putative migration history, effective population sizes, and migration rates among populations across different regions. Populations of extremely small sizes or those located far from the focal regions were excluded from the analysis. In total, 531 individuals from 35 populations across the QTP*ss*, the Himalayas, and the HDM were analyzed. Estimates of directional gene flow among all the samples indicated an asymmetric migration to the Himalayas from the HDM and QTP*ss* (Figure 4a). The HDM populations were the most significant source of migration, with the highest migration rate observed from the HDM to the Himalayas (Nm = 4.18). A secondary high migration rate was noted from the QTP*ss* to the Himalayas (Nm = 1.46). Relatively low migration rates were found from the Himalayas to the HDM and the QTP*ss* (Nm = 0.31 and 0.44, respectively). The migration pattern differed significantly between diploid and tetraploid plants. Migration among tetraploid samples was predominantly asymmetrical, with a higher migration rate from the HDM to both the Himalayas and the QTP*ss* (Nm = 2.91 and 1.60, respectively) and much lower rates in the opposite direction (Nm = 0.23 and 0.01, respectively). In contrast, the migration rates among diploid samples were more comparable, with relatively uniform rates (Nm = 0.78–0.12, with a mean value of 0.38) across regions, indicating a trend toward a more symmetrical and lower migration pattern.

### 2.5. Ecological Niche Modeling

**Figure 5:**
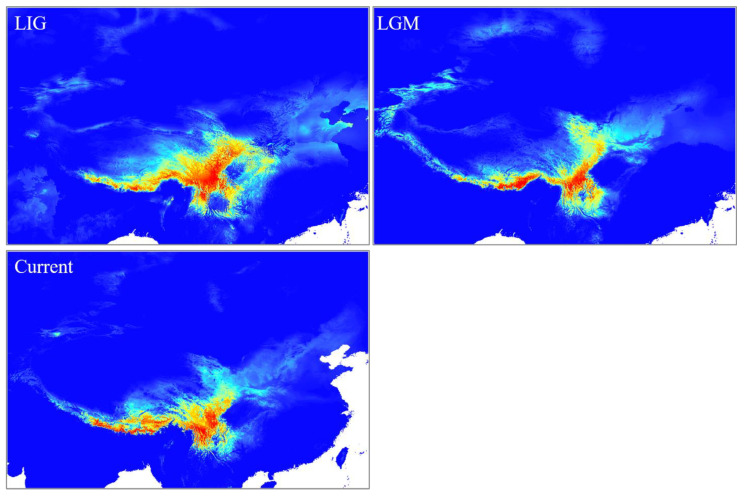
The potential suitable distribution area of *Lepisorus clathratus* during the Last Interglacial (LIG) period, the Last Glacial Maximum (LGM), and the present time, based on ecological niche modeling.

The results of the ecological niche modeling (ENM) indicate that the environmental variables contributing most to model predictions were temperature seasonality (bio4) and the mean temperature of the driest quarter (bio9). All models demonstrated a high predictive performance, with average AUC scores across ten runs ranging from 0.901 to 0.944 across the three periods. The historical distribution patterns of *Lepisorus clathratus* suggest a dynamic response to climatic fluctuations, showing significant contraction during the Last Glacial Maximum (LGM), followed by partial recovery in the present, though not to the full extent of its distribution during the Last Interglacial (LIG) period (Figure 5). During the LIG period, *L. clathratus* had a widely distribution range that primarily spanned the Hengduan Mountains and the southern and eastern regions of the Qinghai–Tibetan Plateau. In contrast, during the LGM, its range contracted significantly, becoming more restricted and fragmented, and was largely confined to the southern portions of the HDM and with some concentration at the southeastern edge of the Qinghai–Tibetan Plateau. In the present day, the distribution of *L. clathratus* has expanded, though not to the same extent observed during the LIG period. The species now occupies a broader range compared with the LGM, being primarily distributed across the HDM and along the southern and southeastern margins of the Qinghai–Tibetan Plateau.

## 3. Discussion

This study provides comprehensive insights into the genetic diversity, geographic distribution, and evolutionary history of *Lepisorus clathratus*, a polyploid fern species endemic to the Himalayan–Hengduan Mountains region. Our findings underscore the significant role of polyploidy in shaping the genetic structure and distribution patterns of plant species in these alpine regions, particularly in response to Quaternary climatic changes.

### 3.1. Polyploidy and the Origins, Establishment, and Expansion of Lepisorus clathratus Tetraploid Populations

The tetraploid populations of *Lepisorus clathratus* very likely have multiple origins, which is a common phenomenon in polyploid plants [22]. Multiple independent origins of polyploidy can occur when diploid populations undergo genome duplication under different environmental conditions or in distinct geographical locations. The findings from both genetic structure analyses and phylogenetic assessments lend support to the hypothesis that tetraploid *L. clathratus* arose through multiple independent tetraploidization events. Specifically, *L. clathratus* polyploids displayed a total of 21 haplotypes dispersed across four distinct genetic groups, which were divided by haplotype network structure and phylogenetic relationships (Figure 2A and Figure S1). It is noteworthy that the ancestral haplotypes of each group underwent tetraploidization. *L. clathratus* probably underwent autotetraploidization, a hypothesis supported by the coexistence and haplotype sharing between polyploids and diploids within a single population. For instance, the GB population in the QTP*ss* contained diploid, tetraploid, and hexaploid individuals that all shared haplotype H37.

The establishment of polyploids of Groups I, III, and IV was limited, likely due to the effects of minority cytotype exclusion (MCE) [23], which would have restricted their population size. As a result, polyploid populations tended to be small and often occupied more marginal, drier habitats compared with diploid populations. Thus, polyploids of these groups tended to be either outcompeted or forced to occupy niches separate from their diploid counterparts. For Group II, the situation was reversed. Diploids of Group II only were only distributed among 5 populations, while polyploids existed in 23 populations. Moreover, plants belonging to Group II exhibited no ploidy variation within populations, except for the small CA population, which contained only one diploid and one polyploid. This suggests that polyploidy likely replaced the ancestral diploid populations within Group II, and that climate change may have driven this process. The results of the ecological niche modeling showed that *L. clathratus* experienced a significant range contraction in the Hengduan Mountains (HDM) during the Last Glacial Maximum (LGM), which has been a primary distribution area for Group II.

Overall, these observations highlight the complex evolutionary dynamics between diploid and polyploid populations in *L. clathratus*. In some cases (Groups I, III, and IV), diploid ancestors appear to have exerted a strong influence, limiting polyploid establishment and distribution. In others (Group II), polyploids have not only survived but also thrived, suggesting potential adaptive advantages or differences in evolutionary trajectories. The establishment of tetraploid populations of *Lepisorus clathratus* could be attributed to their increased ecological tolerance compared with their diploid counterparts. Polyploidy often confers greater environmental resilience, allowing tetraploids to thrive in a broader range of habitats [24]. This could explain the significant presence of tetraploids in the HDM, a region known for its complex topography and microclimatic diversity. Tetraploids might have a competitive advantage in these diverse environments, facilitating their establishment and subsequent expansion. Furthermore, the post-glacial expansion patterns observed in *L. clathratus* [21] align with the distributions of tetraploids in non-focal regions, indicating that tetraploid populations of Group II played a crucial role in recolonizing areas that were previously uninhabitable due to glaciation. However, the demographic history analysis indicates that this group probably underwent population stabilization or contraction. The expansion observed in Group II seems to contradict the results of the demographic history analysis. However, when we consider that demographic expansion is driven by haplotype diversity, it is plausible that diploid haplotypes, impacted by glacial periods, might have undergone significant extinction to a considerable extent. However, polyploids may not inherit all chloroplast haplotypes from their ancestral diploids, as it is likely that only a portion of the diploid ancestors underwent polyploidization. Consequently, this would have led to a decrease in haplotype diversity, ultimately yielding the result of demographic contraction.

### 3.2. Role of Polyploidy in Genetic Structure

Polyploidy may have not only contributed to the species’ ability to colonize new areas but have also shaped the genetic structure among and within populations of *Lepisorus clathratus*. The likely independent origins of polyploid *L. clathratus* in different regions have resulted in distinct polyploid plants with significant genetic differences. These polyploids coexisted with local populations as they migrated across regions, leading to notable genetic differences within the populations. This phenomenon was particularly evident in certain populations from the Himalayas and the eastern Hengduan Mountains (Figure 2B), where these populations often contained polyploids originating from the QTP*ss* or the western Hengduan Mountains. In contrast, the polyploids that migrated across regions also formed independent populations in the Himalayas or the WHDM, exhibiting striking genetic differences when compared with neighboring local populations. The above speculation was supported by the results of the AMOVA analysis, which indicate significant genetic variation both within and between populations (Table 2). Among the focal regions, the Himalayan populations contained the lowest haplotype number but the highest nucleotide diversity (Table 1). This discrepancy also supports the above hypothesis that the immigration of polyploids across regions altered the local genetic structure, as the introduction of alien populations with distinct genetic backgrounds greatly enhanced local genetic diversity and genetic structure, especially in the Himalayan populations.

The cross-regional migration of these polyploids has also altered the genetic structure of *Lepisorus clathratus* on a regional scale. This migration process facilitated the sharing of unique genetic diversity among regions (QTP*ss*, Himalayas, and HDM), reducing genetic differences between them. The likely independent origins and cross-regional migration of these polyploids might promote new genetic variations, which could accumulate more rapidly due to the polysomic masking and genetic recombination typical of polyploids, thereby enhancing the overall genetic diversity of the species [25]. This genetic diversity may provide variations for populations to adapt to rapidly changing environments in the future, thereby ensuring the long-term survival and evolutionary success of the species. On the other hand, the introduction of alien polyploids may exert ecological competitive pressure on local populations, potentially affecting their maintenance. This hypothesis requires further in-depth investigation. Polyploidy has long been recognized as a crucial evolutionary mechanism in plants, enabling species to adapt to diverse and often harsh environments [3,13]. In *Lepisorus clathratus*, the existence of diploid, tetraploid, and hexaploid populations highlights the complexity of polyploid formation and maintenance in natural settings. Our results revealed distinct geographic distributions of these ploidy levels, with tetraploid populations predominantly occupying the HDM and expanding further north and tto wider areas of the non-focal region, such as Xinjiang, Beijing, and Inner Mongolia. Meanwhile, diploids were more prevalent in the QTP*ss* and the eastern part of the HDM, but there were still a few polyploids colonizing arid and minute habitats in the Himalayas. The restricted distribution of polyploid populations in specific habitats suggests that polyploids from the QTP*ss* may confer selective advantages or constraints in certain environments, possibly due to factors like environmental tolerance, reproductive isolation, or ecological niche specialization [26]. Based on field observations, tetraploid and hexaploid plants from the QTP*ss* tend to grow in extremely arid habitats, often resulting in small population sizes, typically consisting of only a few individuals.

### 3.3. Impact of Quaternary Climatic Changes

The Quaternary period, characterized by repeated cycles of glaciation and interglaciation, significantly impacted the distribution and genetic structure of many plant species, including *Lepisorus clathratus* [11]. For *L. clathratus*, our ecological niche modeling (ENM) and phylogeographic analysis suggest that these climatic fluctuations led to significant contractions and expansions in its range, particularly during the Last Glacial Maximum (LGM) and the Last Interglacial (LIG) periods. The genetic data supported a model of post-glacial recolonization, where populations expanded from refugia in response to the retreat of ice sheets, leading to the current distribution patterns. One of the critical factors during this time was the interplay between polyploidy and climatic fluctuations, which influenced the migration patterns and evolutionary trajectories of these species. Our research also supported another model: During the glacial periods, refugia existed for populations in the QTP*ss*. After a glaciation, diploid populations rapidly diversified and expanded locally, limiting the establishment of polyploids in local populations. Populations of *L. clathratus* in the QTP*ss* most likely survived in situ during the LGM, as evidenced by the abundance of haplotypes endemic to these regions. Previous studies indicated that populations in the QTP*ss* diversified prior to the LGM, shifting to microrefugia during glacial periods and subsequently expanding into nearby areas [21]. Our findings align with this hypothesis, as demonstrated by the demographic expansion observed in the mismatch distribution analysis (Figure 3) and the habitat persistence during the LGM indicated by the ecological niche modeling (Figure 5). Furthermore, our results revealed that demographic expansion and genetic diversification primarily occurred in diploid populations, while only a few polyploid individuals contributed to cross-region migration. This pattern of diploid diversification, coupled with a local expansion model in the Group III populations, contrasts with the polyploid replacement and regional expansion model observed in Group II.

Polyploidy, which is the condition of having more than two complete sets of chromosomes, plays a crucial role in the evolutionary success of plant species, particularly in the context of environmental change [26]. During the Quaternary period, polyploid populations of *Lepisorus clathratus* in Group II likely exhibited enhanced adaptability to the shifting climate compared with their diploid counterparts. As a result, these polyploid populations could colonize and thrive in a wider range of habitats, leading to their persistence and expansion during periods of climatic instability. The coupling of polyploidy with Quaternary climatic changes had significant implications for the migration and distribution patterns of *L. clathratus*. The migration patterns of polyploid populations during this period were likely more dynamic and widespread compared with those ofw diploids. This was supported by our findings that polyploid populations of *L. clathratus* tended to undergo long-distance migration, which was particularly evident in the extensive spread of polyploid populations from the HDM to northwestern and northern China. The enhanced migratory capability of polyploids, possibly driven by their increased ecological tolerance, allowed these populations to exploit new habitats as glaciers retreated and new ecological niches became available. The reproductive strategy of intragametophytic selfing in polyploid spores may be another key factor facilitating the long-distance colonization of polyploids. After polyploid formation, reproductive modes often shift, and in homosporous ferns, polyploids can utilize an intragametophytic selfing strategy, allowing them to complete reproduction with a single spore [27]. In contrast, diploids typically rely on outcrossing, which limits their ability to establish populations over long distances. However, this hypothesis requires further evidence for validation in *L. clathratus*. The asymmetrical gene flow observed between regions, with significant migration occurring from the HDM and the QTP*ss* to the Himalayas, further underscores the role of polyploidy in shaping the geographic distribution of *Lepisorus clathratus*. The HDM, with its complex topography and diverse microhabitats, likely served as a crucial refugium for polyploid populations during glacial maxima [28]. As the climate warmed and glaciers receded, these polyploid populations would have been among the first to expand into newly available habitats, driving the recolonization of areas like northwestern and northern China and the Himalayas. The genetic diversity observed in these regions today reflects the historical movements and expansion of these polyploid populations during the Quaternary. The significance of these polyploid migration patterns lies in their contribution to the current genetic diversity and structure of *L. clathratus*. Polyploids, by virtue of their greater genetic variability and ecological flexibility, not only survived the climatic upheavals of the Quaternary but also facilitated gene flow between otherwise isolated populations. This gene flow, coupled with the expansion of polyploid populations into new ecological niches, was likely one of the key mechanisms allowing polyploids to maintain their populations.

## 4. Materials and Methods

### 4.1. Samples

A total of 66 populations with 586 individuals were sampled throughout the species’ distribution range, which included three focal regions—the Himalayas, the Qinghai–Tibetan Plateau sensu stricto (QTP*ss*), and the Hengduan Mountains (HDM)—and their peripheral mountainous region of western and northern China (Figure 1, Table 1). At each site, we collected plants from individuals that were at least 10 m apart to avoid collecting clones originating from the same rhizome. Usually, only a few individuals were obtained from each population due to the small population size of this species in the wild. The sample size per population ranged from 1 to 51 individuals, with an average of 9 per locality (Table 1). Fresh leaves for DNA extraction were obtained and preserved in silica gel. Some living plants were transplanted into a greenhouse at the Institute of Botany, Chinese Academy of Sciences. All vouchers were deposited in PE, Institute of Botany, Chinese Academy of Sciences.

### 4.2. Determination of Ploidy Levels

Flow cytometry (FCM) is a convenient method to detect the variation of the ploidy level in large samples [29]. We conducted FCM using fresh leaves or silica-gel-dried materials to estimate the DNA content (2C DNA value), mainly following the protocol described in our previous study [30]. We macerated approximately 1 to 2 cm^2^ of leaves per individual by chopping the material with a new razor blade in a Petri dish containing 1 mL of fresh ice-cold Otto I buffer (0.1 mol/L citric acid monohydrate, 0.5% (*v*/*v*) Tween-20, pH of 2–3). We filtered the macerated material through a 50 µm nylon mesh and centrifuged it at 150 g for 5 min. Following centrifugation, we removed the supernatant and resuspended the pellet in 100 µL of fresh ice-cold Otto I buffer and 200 µL of Otto II buffer (0.4 mol/L Na_2_HPO_4_ 12H_2_O). We stained the suspension with 50 µg/mL propidium iodide and 50 µg/mL RNase and used it for FCM measurements on an Elite flow cytometer (BD FACSCalibur, USA). We used *Zea mays* ssp. *mays* B73 (2C = 5.64 pg) as an internal reference [31]. The relationship between 2C values and ploidy levels was inferred based on the DNA content of one individual of diploid *L. waltonii* with a known chromosome number (2n = 72).

### 4.3. DNA Extraction, Polymerase Chain Reaction Amplification, and DNA Sequencing

We extracted total DNA from silica-gel-dried leaves using a Plant Genomic DNA Kit (Tiangen Biotech, Beijing, China). We amplified four cpDNA regions, *matK*, *psbZ*, *rpl32-trnP* and *trnT*, using primers reported by Zhao et al. (2019) [32]. We amplified all four cpDNA regions using a standard polymerase chain reaction (PCR) consisting of the following steps: pre-denaturation at 94 °C for 4 min; 35 cycles of denaturation at 94 °C for 30 s, annealing at 55–60 °C for 30 s (55 °C for *trnT*, 58 °C for *matK*, and 60 °C for *rpl32-trnP* and *psbZ*), and extension at 72 °C for 90 s; and a final extension at 72 °C for 10 min. We verified successful amplification by visualizing the PCR products on a 1% agarose gel in TAE buffer. We purified the products using a TIANgel Midi Purification Kit (Tiangen Biotech, Beijing, China) and directly sequenced them on an ABI 3700 automated DNA sequencer (Applied Biosystems, Foster City, CA, USA). All DNA sequences were deposited in GenBank (MK484715-MK486766).

### 4.4. Data Analysis

We aligned all the sequences using MAFFT version 7 [33] with the FFT-NS-2 algorithm and adjusted the results in BioEdit v.7.7.1 [34]. We concatenated the four cpDNA regions into a single matrix, and we carried out chloroplast haplotype assignment in DnaSP v.5.10 [35]. The haplotype diversity (*Hd*), nucleotide diversity (π), and the number of haplotypes (*h*) were calculated using Arlequin v.3.5 [36] and DnaSP [35].

To study the genetic relationships between different ploidies and among populations, phylogenetic trees were constructed using two methods, maximum likelihood (ML) and Bayesian inference (BI), using IQ-tree v.1.6 [37] and MrBayes v.3.2 [38,39], respectively. We used ModelFinder [40], implemented in the IQ-tree software, to infer the appropriate nucleotide substitution model for phylogenetic analysis. The ML trees were generated in IQ-TREE [37] with 1000 ultrafast bootstrap replicates [41]. ML support values (ultrafast bootstrap, UFBoot) of each node were checked in FigTree v.1.4 [42]. In MrBayes, we conducted two simultaneous independent runs of four Markov Chain Monte Carlo (MCMC) chains, each from a random starting tree. The runs consisted of 10,000,000 generations, with sampling every 1000 generations. We checked for convergence of the simultaneous runs in Tracer v.1.5 [43] and determined the suitability of a 25% burn-in. We determined the posterior probabilities (PPs) based on a majority-rule consensus tree of all the sampled trees following a 25% burn-in. In addition, to investigate the relationship among haplotypes, a statistical parsimony haplotype network with median-joining (MJ) was built using Network v.5.0 [44], and each indel was treated as a single mutation event.

Analysis of molecular variance (AMOVA) and fixation indices (FST) were calculated using Arlequin [36]. The significance of the test was assessed using 1000 permutations of each pairwise comparison. Historical population dynamics was investigated using Tajima’s *D* [45], Fu’s *Fs* [46], and mismatch distributions of pairwise differences [47]. Tajima’s *D* and Fu’s *Fs* were implemented in Arlequin, with the deviation from neutrality determined from 1000 coalescent simulations. To test the population expansion hypothesis, mismatch distributions of pairwise sequences were calculated using Arlequin with 1000 bootstrap replicates.

To assess the historical migration pattern of *Lepisorus clathratus*, we estimated the gene flow between the QTP*ss*, the Himalayas, and the HDM using the software package MIGRATE v.4.4.3 [48]. The full migration model was conducted through a long chain of 1 × 105 recorded genealogies with a sampling increment of 100 iterations and a 1 × 104 burn-in. To improve the Markov Chain Monte Carlo searches, an adaptive heating scheme with four chains (with temperatures of 1, 1.5, 3 and 1,000,000) and a swapping interval of 1 was applied. The equation 4Nm = θ × M was employed to compute the number of migrations per generation (Nm) among the populations.

To explore the suitable distribution range of *Lepisorus clathratus*, we conducted ecological niche modeling (ENM) via maximum entropy using Maxent v.3.4.3 with default settings [49]. The current distribution information of *L. clathratus* was obtained from our field investigations and herbarium collections of PE. A set of 19 bioclimatic variables at a 2.5 arc-min resolution covering the distribution range was downloaded from the WorldClim database (www.worldclim.org, accessed on 1 September 2024). Six uncorrelated (|r| < 0.7) bioclimatic variables (bio4 Temperature Seasonality, bio7 Temperature Annual Range, bio9 Mean Temperature of Driest Quarter, bio12 Annual Precipitation, bio15 Precipitation Seasonality, and bio17 Precipitation of Driest Quarter) were selected for the final analysis. Outputs were set at 25% for testing and 75% for training in the model. A model prediction was considered useful when the area under the curve (AUC) score was in the range of 0.7–0.9, and good when >0.9 [50]. We set the maximum number of iterations to 500 and the number of replicates to ten. To identify potential historical distribution changes, we projected ENMs to different historical periods with Maxent. Paleoclimatic data with a resolution of 2.5′, which included the Last Glacial Maximum (LGM) [51], and the Last Interglacial (LIG) period [52], were obtained from the paleoclim database (http://www.paleoclim.org, accessed on 1 September 2024).

## 5. Conclusions

The *Lepisorus clathratus* exemplifies the intricate interplay between polyploidy, genetic diversity, and environmental adaptation in alpine plant species. Our study highlights the crucial role of polyploidy in shaping the genetic diversity, geographic distribution, and evolutionary success of *L. clathratus* in the Himalayan–Hengduan Mountains regions. Polyploid populations, particularly tetraploids, have demonstrated enhanced ecological flexibility and a greater ability to colonize new environments compared with diploids. The results indicate that polyploidy facilitated the post-glacial expansion of *L. clathratus*, possibly allowing these populations to exploit diverse habitats in the aftermath of the Last Glacial Maximum. Furthermore, the analysis of genetic structure and gene flow reveals significant regional differentiation, with polyploids showing higher migration rates, which likely contributed to their broader distribution. These findings underscore polyploidy’s pivotal role in plant adaptation to historical climatic changes and emphasize its importance in shaping the genetic structure across complex landscapes.

## Figures and Tables

**Table 1:** Genetic diversity parameters of *Lepisorus clathratus* in focal and non-focal regions. In this table, *h* represents the haplotype number, *Hd* represents the haplotype diversity, and π represents the nucleotide diversity.

Region	*h*	*Hd*	π
Himalaya	15	0.8418	0.00524
QTPss	19	0.7143	0.00053
WHDM	19	0.6927	0.00224
EHDM	20	0.7819	0.00249
HDM	34	0.7489	0.00265
Non-focal regions	13	0.7305	0.00052
All	70	0.8991	0.00332

**Table 2:** AMOVA results for *Lepisorus clathratus* based on grouping by focal regions and ploidy levels.

Data Grouping	Source of Variation	d.f.	Sum of Squares	Variance Components	Percentage of Variation	Fixation Indices
The three focal regions ^#^	Among regions	3	996.539	2.17516	30.65	FSC: 0.51608 **
	Among populations within regions	41	1136.605	2.53977	35.79	FST: 0.66441 **
	Within populations	466	1109.773	2.38149	33.56	FCT: 0.30652 **
	Total	510	3242.918	7.09642		
The three ploidy levels	Among ploidies	2	390.265	0.71395	6.38	FSC: 0.81403 **
	Among populations Within ploidies	54	3536.334	8.5237	76.21	FST: 0.82590 **
	Within populations	391	761.407	1.94733	17.41	FCT: 0.06383
	Total	447	6598.527	13.9605		

^#^: The three focal regions considered in this AMOVA analysis are the Himalayas, QTP*ss* (Qinghai–Tibetan Plateau sensu stricto), and the HDM (Hengduan Mountains). The HDM, was further subdivided into two subregions: WHDM (sestern Hengduan Mountains) and EHDM (eastern Hengduan Mountains). **: *p* ≤ 0.001.

**Table 3:** Results of the neutrality tests.

Group	Number of Sequences	Tajima’s *D* ^#^	*p*-Value	Fu’s *Fs* ^#^	*p*-Value
Group I	70	0.15261	0.598	6.34018	0.987
Group II	315	−1.60835	0.016	−4.23654	0.155
Group III	148	−1.863	0.008	−13.16619	0
Group IV	53	−1.72918	0.022	−7.80749	0

^#^: Tajima’s *D* evaluates the disparity between the count of segregating sites and average pairwise nucleotide differences, with negative values suggesting an abundance of low-frequency variants that may indicate positive selection or population expansion, while Fu’s *Fs* detects an excess of rare alleles within a population, potentially implying recent growth or positive selection.

## Data Availability

Source data are available from the authors upon request.

## Supplementary Materials

The following supporting information can be downloaded at: https://www.mdpi.com/article/10.3390/plants13223181/s1, Figure S1: Majority consensus tree of haplotypes obtained in the Bayesian analysis based on the combined molecular dataset, which included sequences of *matK*, *psbZ*, *rpl32-trnP*, and *trnT*. Numbers of each branch are support values in the order of MPJK/UFBoot/BIPP. The node with MPJK < 50%, UFBoot < 80%, and BIPP < 0.8 was not shown; Figure S2: Composition of haplotype groups in *Lepisorus clathratus* populations within non-focal and focal regions. (A) All populations of *L. clathratus* populations in the non-focal region belonged exclusively to Group II. (B) Population-specific haplotype group composition of *L. clathratus* populations in the focal region. The size of the circles represents the number of individuals in each population, and the color of the pies indicates the haplotype group: green for Group I, blue for Group II, white for Group III, and gray for Group IV; Table S1: Geographic information, ploidy level composition, and genetic diversity parameters of the populations. This table includes the number of individual numbers (no.), ploidy levels (2X: diploid; 4X: tetraploid; 6X: hexaploid; xX: ploidy level unknown), haplotype number (*h*), haplotype diversity (*Hd*), and nucleotide diversity (π) for each population; Table S2: Estimates of historical gene flow using MIGRATE v.4.4.3. This table presents data analyzed using all the samples, the 2X diploid samples and the 4X tetraploid samples. The median and 95% credibility interval provide statistical data regarding the migration rate, while Nm represents the number of individuals that migrate per generation.
