# Supplementary material for: The Role of Polyploidy in the Genetic Structure and Expansion of Lepisorus clathratus in the Qinghai–Tibetan Plateau and Hengduan Mountains
Source: Plants (Basel). 2024 Nov 13;13(22):3181. doi: 10.3390/plants13223181 (PMC11597778; doi:10.3390/plants13223181)
Supplement: Supplementary file 1 [file plants-13-03181-s001.zip › plants-3232250-supplementary.pdf]

Figure S1 Majority consensus tree of haplotypes obtained in Bayesian analysis based on the combined molecular dataset including sequences of *matK*, *psbZ*, *rpl32-trnP* and *trnT*. Numbers of each branch are support values in the order of MPJK/UFBoot/BIPP. The node with MPJK < 50%, UFBoot < 80% and BIPP < 0.8 was not shown.

Figure S2. Composition of haplotype groups in *Lepisorus clathratus* populations within non-focus and focal regions. (A) All populations of *L. clathratus* populations in the non-focus region belonged exclusively to Group II. (B) Population-specific haplotype group composition of *L. clathratus* populations in the focal region, the size of the circles represents the number of individuals in each population, and the color of the pies indicates the haplotype group: green for Group I, blue for Group II, white for Group III, and gray for Group IV.

Table S1 Geographic information, ploidy level composition, and genetic diversity parameters of populations. This table includes individual number (no.), ploidy levels (2X diploid, 4X tetraploid, 6X hexaploid, xX ploidy level unknown), haplotype number (*h*), haplotype diversity (*Hd*), and nucleotide diversity (π) for each population.

Table S2 Estimates of historical gene flow using MIGRATE. This table presents data analyzed with all samples, 2X diploid samples and 4X tetraploid samples. The Median and 95% credibility interval provide statistical data regarding the migration rate, while Nm represents the number of individuals that migrate per generation.

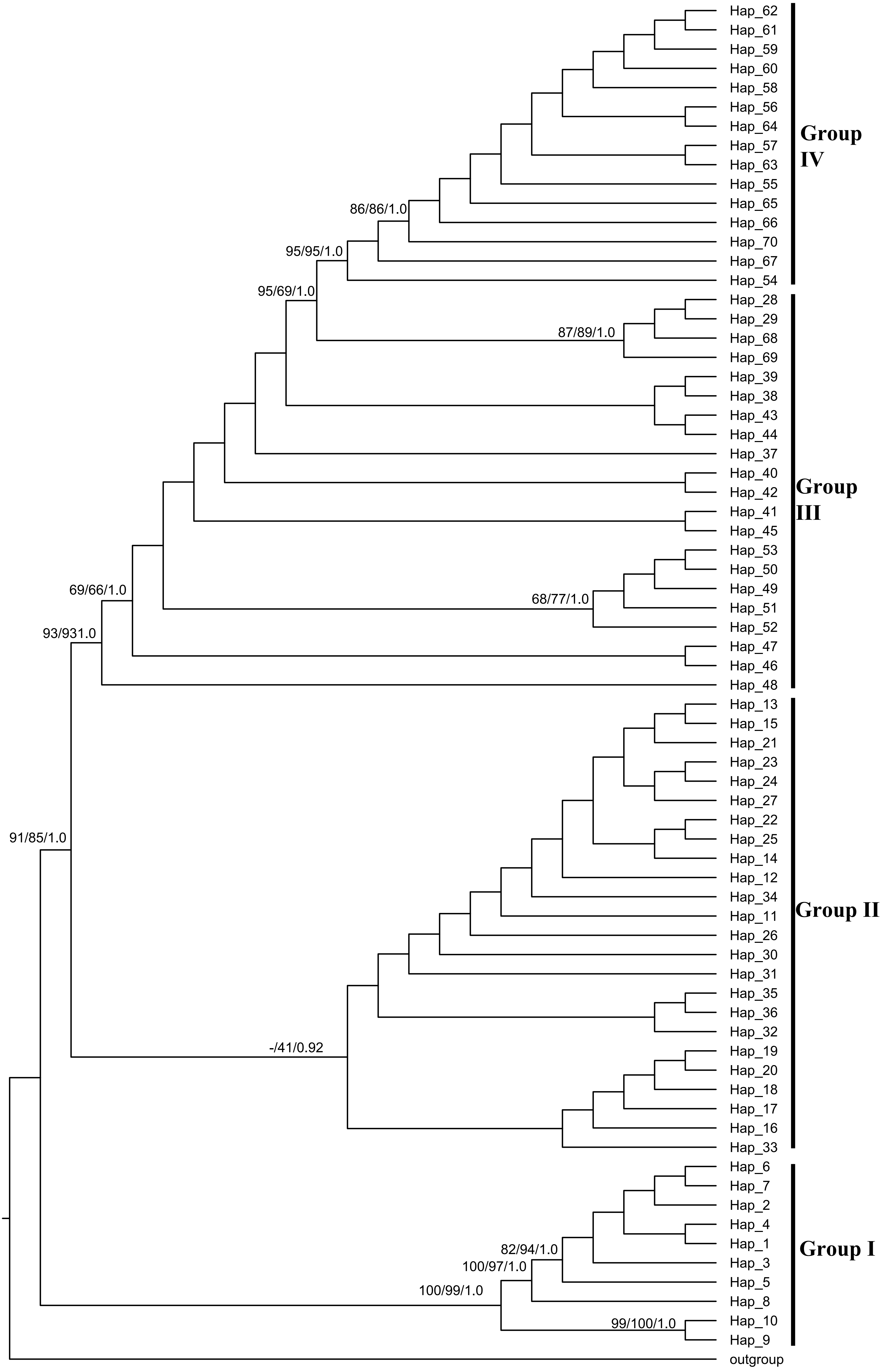
Hap_62
Hap_61
Hap_59
Hap_60
Hap_58
Hap_56
Hap_64
Hap_57
Hap_63
Hap_55
Hap_65
Hap_66
Hap_70
Hap_67
Hap_54
Group IV
86/86/1.0
95/95/1.0
95/69/1.0
Hap_28
Hap_29
Hap_68
Hap_69
87/89/1.0
Hap_39
Hap_38
Hap_43
Hap_44
Hap_37
Hap_40
Hap_42
Hap_41
Hap_45
Hap_53
Hap_50
Hap_49
Hap_51
Hap_52
68/77/1.0
Hap_47
Hap_46
Hap_48
Group III
69/66/1.0
93/931.0
91/85/1.0
Hap_13
Hap_15
Hap_21
Hap_23
Hap_24
Hap_27
Hap_22
Hap_25
Hap_14
Hap_12
Hap_34
Hap_11
Hap_26
Hap_30
Hap_31
Hap_35
Hap_36
Hap_32
Hap_19
Hap_20
Hap_18
Hap_17
Hap_16
Hap_33
Group II
-/41/0.92
Hap_6
Hap_7
Hap_2
Hap_4
Hap_1
Hap_3
Hap_5
Hap_8
Hap_10
Hap_9
Group I
82/94/1.0
100/97/1.0
100/99/1.0
99/100/1.0
outgroup

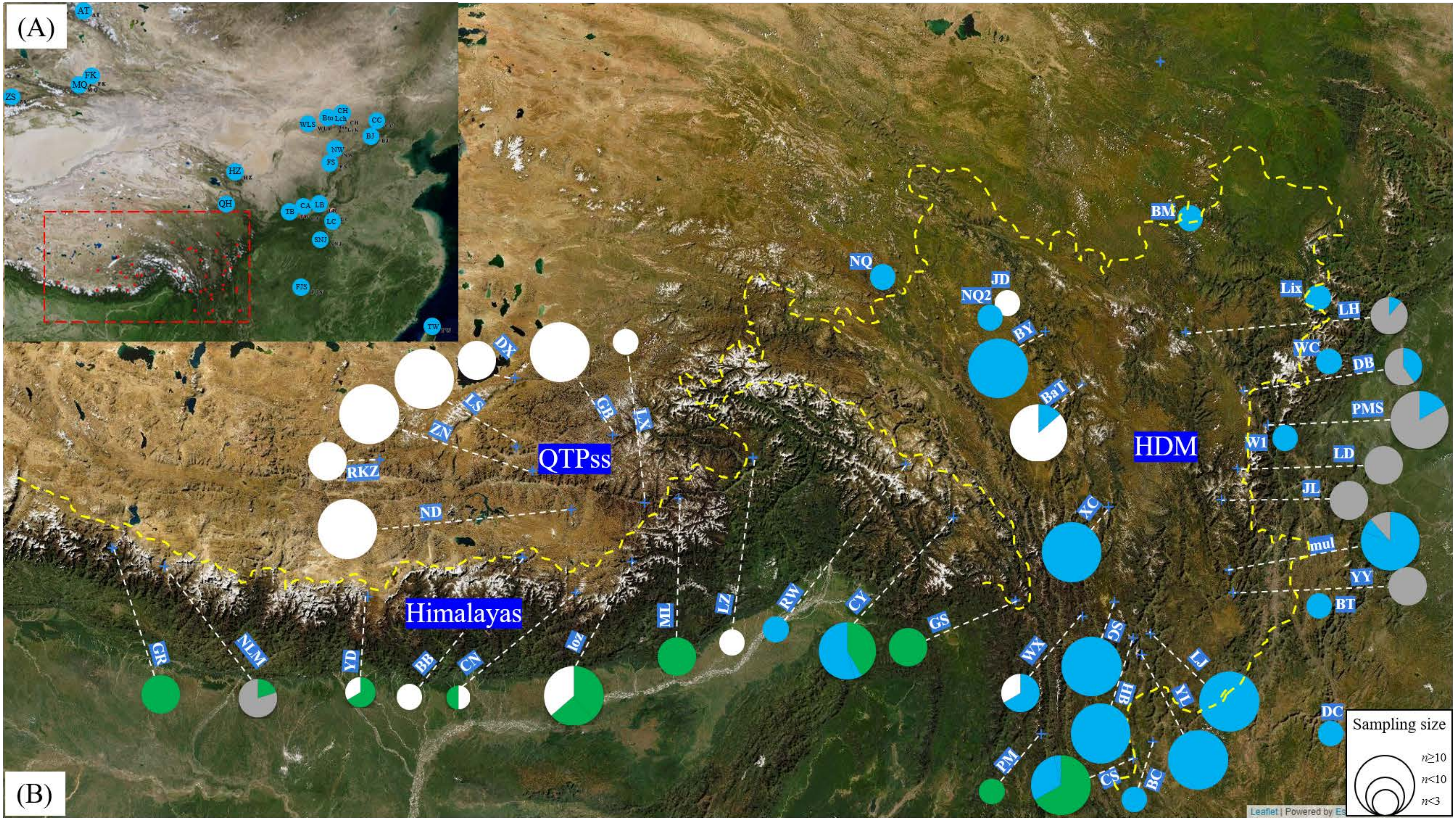

(A)
(B)
AT
FK
MQ
ZS
WLS
Bto
Lch
CH
CC
BJ
NW
FS
HZ
QH
TB
CA
LB
LC
SNJ
FJS
TW
DX
LS
ZN
GB
LX
RKZ
QTPss
ND
Himalayas
GR
NLM
YD
BB
CN
loz
ML
LZ
RW
CY
GS
NQ
JD
NQ2
BY
BaT
BM
HDM
Lix
LH
WC
DB
PMS
W1
LD
JL
XC
mul
YY
BT
WX
SG
HB
YL
LJ
PM
CS
BC
DC
Sampling size
n≥10
n<10
n<3
Leaflet | Powered by Es

Region	Population codes	Longitude	Latitude	Altitude	no.	2X	4X	6X	xX	haplotype constitution	*h*	*Hd*	π*10-5
Himalayas	CN	92.00033	27.89205	4040	2	1	1			H8 H37	2	1	715
	BB	91.21985	28.24389	4110	2		2			H37	1	0	0
	CY	97.52	28.78	3400	24		2		22	H1 H4 H16 H18 H32	5	0.7101	384
	GR	85.21667	28.46667	3100	4	2			2	H6 H7	2	0.5	19
	loz	92.76307	28.27848	4000	33	21	1	5	6	H1 H3 H5 H9 H10 H37 H49	7	0.7557	482
	NLM	85.98451	28.16321	3700	5		4		1	H1 H67	2	0.4	391
	YD	88.97515	27.7574	3500	3	1	1		1	H1 H37	2	0.667	554
	GS	98.35	27.73	3500	8	8				H1	1	0	0
	LZ	94.57356	29.56637	3750	1		1			H37	1	0	0
	ML	93.4444	29.1027	3100	4	4				H1	1	0	0
	RW	96.75453	29.50446	4600	1				1	H16	1	0	0
Qinghai-Tibetan Plateau *sensu stricto*	GB	92.48	29.89	4260	34	29	2	3		H28 H29 H37 H39 H42 H43 H46 H49 H50 H68 H69	11	0.8004	67
	DX	91.1141	30.5908	4600	3				3	H37	1	0	0
	LS	91.09942	29.71303	4100	28	19	2	2	5	H37 H44 H45 H46 H49 H51 H52	7	0.6958	49
	ND	91.89293	28.91734	4470	26	20			6	H37 H40 H41 H46 H47 H49 H53	7	0.7415	54
	ZN	91.31748	29.37506	4200	12		11	1		H37 H46	2	0.1667	6
	LX	93.07	29.05	3200	1				1	H69	1	0	0
	RKZ	89.11501	29.54564	4000	6			6		H37	1	0	0
Hengduan Mountains – the western Hengduan Mountains	BC	100.3977	25.9426	3100	1				1	H32	1	0	0
	BaT	99.25877	30.50756	3700	22		22			H20 H37 H38	3	0.3247	103
	BY	98.82	31.21	3600	27		26		1	H12 H16 H20	3	0.416	58
	JD	98.22	31.5	3500	1				1	H48	1	0	0
	CS	100.104	25.6895	3200	27	3	8		16	H1 H2 H22	4	0.7037	356
	HB	100.1087	27.35116	3800	7		7			H32	2	0.2857	32
	SG	99.8135	27.7983	3350	38		29		9	H16 H17 H19 H21 H32 H34 H35	7	0.606	53
	LJ	100.35	27.36	3300	15	15				H32	1	0	0
	WX	99.3695	27.6306	3200	3				3	H22 H25 H37	3	1	225
	XC	99.7502	28.9766	4150	30	30				H16 H32 H33	3	0.131	7
	NQ	96.51667	31.88333	3790	1				1	H12	1	0	0
	NQ2	98.07	31.38	3500	1				1	H12	1	0	0
	PM	98.68366	25.97293	3150	1	1				H8	1	0	0
	YL	100.2634	27.0283	3800	33		32		1	H32	1	0	0
Hengduan Mountains – the eastern Hengduan Mountains	BM	100.93	32.68	3500	2				2	H12	1	0	0
	BT	102.8156	27.75161	2700	2		2			H21	1	0	0
	DC	103.2	26.1	3000	1				1	H32	1	0	0
	Lix	102.8202	31.6408	2550	1		1			H12	1	0	0
	DB	101.631	30.509	3550	5	4			1	H31 H55 H64 H66	4	0.9	275
	JL	101.4805	29.16787	3200	7	7				H55 H62	2	0.2857	21
	LD	101.65	29.48	3500	6	1			5	H55 H57 H66	3	0.7333	20
	LH	100.8546	31.2006	3000	9				9	H20 H54 H55 H59 H60 H61	6	0.8889	210
	mul	101.45	28.08	3000	51	5	42		4	H21 H32 H33 H55	4	0.3718	97
	PMS	101.96	30.04	2800	12	5			7	H31 H55 H58 H64 H65 H66 H70	7	0.9091	153
	W1	102.27	29.87	3000	1		1			H21	1	0	0
	WC	102.9658	30.8786	3550	1				1	H21	1	0	0
	YY	101.4858	27.8073	3200	9	9				H55 H56 H63	3	0.5556	37
non-focus region	QH	100.5	34.6129	3500	7		6		1	H12	1	0	0
	HZ	102.2575	37.03611	2870	10		10			H27	2	0.2	15
	CA	108.77	33.81	2000	2	1	1			H22	1	0	0
	LB	110.4781	34.4201	2000	2				2	H12	1	0	0
	LC	111.6413	33.7218	2000	1				1	H22	1	0	0
	SNJ	110.68	31.74	2500	2				2	H15	1	0	0
	TB	107.76	33.96	2300	18	15			3	H11 H12 H13 H14 H22 H23 H26	7	0.7843	51
	BJ	115.5894	39.82	1950	4		4			H16	1	0	0
	Bto	111.39	40.82	2000	7		2		5	H16	1	0	0
	CC	115.9	40.9	2000	1				1	H16	1	0	0
	FS	111.55	37.87	2700	1		1			H24	1	0	0
	Lch	112.31	40.61	2000	1				1	H24	1	0	0
	NW	111.93	38.73	2780	1		1			H24	1	0	0
	WLS	109.4	40.7	2000	1				1	H24	1	0	0
	CH	112.53	41.14	2000	2				2	H16 H24	2	1	113
	AT	87.55	48.52	2000	1				1	H12	1	0	0
	MQ	87.14694	43.41389	2200	4		3		1	H12	1	0	0
	FK	88.14	43.89	2000	7		3		4	H12 H24	2	0.4762	35
	ZS	80.54	42.66	2000	1		1			H24	1	0	0
	FJS	108.84	27.7	2100	1		1			H21	1	0	0
	TW	121.43	24.36	3000	1				1	H30	1	0	0

Ploidy level	From	To	Median	95% credibility interval	Nm
all	QTPss	Himalayas	62.33333	21.33333~56.66667	1.45672992
	HDM	Himalayas	85.66667	19.33333~138	4.1831035
	Himalayas	QTPss	11.66667	0~32	0.19716672
	HDM	QTPss	9	0~25.33333	0.43947
	Himalayas	HDM	18.33333	0~42.66667	0.30983328
	QTPss	HDM	16.33333	0~42	0.38170992
2X	QTPss	Himalayas	42.33333	0~172.66667	0.28363331
	HDM	Himalayas	82.33333	2~49.33333	0.45283332
	Himalayas	QTPss	43.66667	4~50	0.11790001
	HDM	QTPss	36.33333	0~75.33333	0.19983332
	Himalayas	HDM	287.6667	134~472.6667	0.77670009
	QTPss	HDM	64.33333	6~64	0.43103331
4X	QTPss	Himalayas	355	128.6667~493.3333	0.3195
	HDM	Himalayas	297.6667	48.6667~488.6667	2.90820366
	Himalayas	QTPss	424.3333	242.6667~499.3333	0.46676663
	HDM	QTPss	163.66667	0~324	1.59902337
	Himalayas	HDM	211	1.33333~31.33333	0.2321
	QTPss	HDM	15.66667	0~32.66667	0.0141
